# Supplementary material for: How Do Flemish Laying Hen Farmers and Private Bird Keepers Comply with and Think about Measures to Control Avian Influenza?
Source: Vet Sci. 2024 Oct 5;11(10):475. doi: 10.3390/vetsci11100475 (PMC11512282; doi:10.3390/vetsci11100475)
Supplement: Supplementary file 1 [file vetsci-11-00475-s001.zip › vetsci-3137299-supplementary.pdf]

Survey private bird keepers

Dear Sir/Madam,

This questionnaire is being used to investigate the welfare implications related to the most recent avian flu measures (confinement requirement) and potential alternatives to these measures.

This survey is particularly useful for individuals and hobbyists with poultry and other birds, providing you the opportunity to be heard. On one hand, your input is crucial in the context of reducing infection and the spread of avian flu in Flanders. This will help map out the usefulness of the confinement requirement and possible alternatives for poultry and other birds. On the other hand, this allows ILVO (Institute for Agricultural, Fisheries, and Food Research) to conduct research that aligns with current concerns. To ensure the data is representative, it is important that as many individuals and hobbyists as possible participate in the survey. Only a large response will yield accurate and representative information. You would greatly help us by filling out this survey, and we would also appreciate it if you could share it with other hobbyists and individuals.

There are no right or wrong answers in this survey; what matters is your opinion. Naturally, we assure you that all information will be treated confidentially. All information is used solely for research purposes.

FREE-RANGE

Question 1: What poultry/farmyard animals or other birds do you keep? And how many of each?

Question 2: Please indicate which of the following options applies to you. Free-range = poultry/other birds can roam inside and outside.

- I have a free-range area for (some of) my birds.
- I do not have a free-range area.

Question 3: Please rate the following measures according to (scale from 1 = no compliance/not effective to 5 = full compliance/most effective)

(a) how meaningful/effective you find them,

(b) whether you have complied with them.

1) Compulsory confinement of poultry or other captive birds to prevent contact with wild birds by keeping them indoors or by
2) Covering outdoor ranges with nets (mesh size of max. 10 cm)
3) Obligation to feed and water poultry and other captive birds indoors or by other means that prevent contact with wild birds
4) A ban on feeding or providing water to poultry and other captive birds from surface water supplies or rainwater to which wild birds have access unless the water has been treated to inactivate viruses

Question 4: Please rate the following measures according to (scale from 1 = no compliance to 5 = full compliance)

(c) whether you think fellow individuals/hobbyists who keep birds have complied with them,

(d) whether professional poultry farmers have complied with them.

1) Compulsory confinement of poultry or other captive birds to prevent contact with wild birds by keeping them indoors or by
2) Covering outdoor ranges with nets (mesh size of max. 10 cm)
3) Obligation to feed and water poultry and other captive birds indoors or by other means that prevent contact with wild birds
4) A ban on feeding or providing water to poultry and other captive birds from surface water supplies or rainwater to which wild birds have access unless the water has been treated to inactivate viruses

Question 5: To what extent did the confinement had a negative or positive effect on… to (scale from 1 = negative to 5 = positive)

1) Workload
2) The breeding
3) The welfare of hobby poultry/other kept birds

Question 6: To what extent did the following animal welfare problems decrease or increase during the confinement obligation? (scale from 1 = decrease to 5 = increase)

1) Prevention of diseases
2) Feather pecking
3) Cannibalism
4) Stress and frustration in hobby poultry/other kept birds
5) Red mite
6) Mortality in hobby poultry/other kept birds
7) Workload
8) Foot dermatitis, hock dermatitis, and other foot injuries

Question 7: To what extent did the following animal welfare problems increase or decrease during the confinement obligation? (scale from 1 = deterioration to 5 = improvement)

1) Bedding quality
2) Indoor climate
3) Condition of feathers, comb, wattles, and body

Question 8: Do you have any other examples of effects on the welfare of hobby poultry/other birds you keep? If so, which ones?

Question 9: How did you implement the confinement obligation?

Please fill in 'Enter your comments here:' with your reasons.

- Covering the free-range with nets
- Indoor confinement
- I did not confine my poultry or other birds and did not cover my free-range area with nets

Question 10: Do you, as a private individual/hobbyist, consider it good or bad that placing nets over the range was also permitted instead of confining the birds indoors? (scale from 1 = bad to 5 = good)

Question 11: How feasible do you think it is to install nets over the range for hobby poultry/other birds you keep? (scale from 1 = not feasible to 5 = feasible)

Question 12: Are you in favor of or against the following alternative measures against avian influenza? (scale from 1 = against to 5 = in favor)

1) Alternative 1: Mandate vaccination of poultry against avian influenza
2) Alternative 2: Ban free-range systems
3) Alternative 3: Reduce poultry density in the region
4) Alternative 4: Culling (with compensation) in case of infection

Question 13: For which target group/region is this applicable?

(1) Alternative 1: Mandate vaccination of poultry against avian influenza and (2) Alternative 2: Ban free-range systems (scale from 1 = not applicable to 5 = applicable)

1) All poultry/kept birds
2) Only professional companies
3) Only private individuals/hobbyists
4) Only in (waterfowl-rich) risk zones

Question 14: For which target group/region is this applicable?

(3) Alternative 3: Reduce poultry density in the region and (4) Alternative 4: Culling (with compensation) in case of infection (scale from 1 = not applicable to 5 = applicable)

5) All poultry/kept birds
6) Only professional companies
7) Only private individuals/hobbyists
8) Only in (waterfowl-rich) risk zones

Question 15: (a) Please indicate which of the following methods you would apply to reduce the risk of infection from wild (water)birds in free-range systems. (b) Please indicate to what extent the following methods would reduce the risk of infection from wild (water)birds in free-range systems. (scale from 1 = reduction to 5 = no reduction)

1) Dense vegetation
2) Deterrents (e.g., lasers, aversive sounds, scarecrows, other animals, etc.)
3) Other

NO-FREE RANGE

Question 1: What poultry/farmyard animals or other birds do you keep? And how many of each?

Question 2: Please indicate which of the following options applies to you. Free-range = poultry/other birds can roam inside and outside.

- I have a free-range area for (some of) my birds.
- I do not have a free-range area.

Question 3: Please rate the following measures according to (scale from 1 = no compliance/not effective to 5 = full compliance/most effective)

(a) how meaningful/effective you find them,

(b) whether you have complied with them.

5) Compulsory confinement of poultry or other captive birds to prevent contact with wild birds by keeping them indoors or by
6) Covering outdoor ranges with nets (mesh size of max. 10 cm)
7) Obligation to feed and water poultry and other captive birds indoors or by other means that prevent contact with wild birds

8) A ban on feeding or providing water to poultry and other captive birds from surface water supplies or rainwater to which wild birds have access unless the water has been treated to inactivate viruses

Question 4: Please rate the following measures according to (scale from 1 = no compliance to 5 = full compliance)

(c) whether you think fellow individuals/hobbyists who keep birds have complied with them,

(d) whether professional poultry farmers have complied with them.

5) Compulsory confinement of poultry or other captive birds to prevent contact with wild birds by keeping them indoors or by
6) Covering outdoor ranges with nets (mesh size of max. 10 cm)
7) Obligation to feed and water poultry and other captive birds indoors or by other means that prevent contact with wild birds
8) A ban on feeding or providing water to poultry and other captive birds from surface water supplies or rainwater to which wild birds have access unless the water has been treated to inactivate viruses

Question 5: To what extent do you think the confinement obligation for private individuals/hobbyists with free-range animals has had a negative or positive effect on… to (scale from 1 = negative to 5 = positive)

4) Workload
5) The breeding
6) The welfare of hobby poultry/other kept birds

Question 6: To what extent do you think that the following animal welfare problems among private individuals/hobbyists with free-range animals have increased or decreased during the confinement obligation? (scale from 1 = decrease to 5 = increase)

9) Prevention of diseases
10) Feather pecking
11) Cannibalism
12) Stress and frustration in hobby poultry/other kept birds
13) Red mite
14) Mortality in hobby poultry/other kept birds
15) Workload
16) Foot dermatitis, hock dermatitis, and other foot injuries

Question 7: To what extent do you think that the following animal welfare problems among private individuals/hobbyists with free-range animals have increased or decreased during the confinement obligation? (scale from 1 = deterioration to 5 = improvement)

4) Bedding quality
5) Indoor climate
6) Condition of feathers, comb, wattles, and body

Question 8: Do you have any other examples of effects on the welfare of hobby poultry/other birds you keep? If so, which ones?

Question 9: If you had free-range animals, would you cover the range with nets or would you confine the birds indoors?

Please fill in 'Enter your comments here:' with your reasons.

- Covering the free-range with nets
- Indoor confinement

Question 10: Do you, as a private individual/hobbyist, consider it good or bad that placing nets over the range was also permitted instead of confining the birds indoors? (scale from 1 = bad to 5 = good)

Question 11: How feasible do you think it is to install nets over the range for hobby poultry/other birds you keep? (scale from 1 = not feasible to 5 = feasible)

Question 12: Are you in favor of or against the following alternative measures against avian influenza? (scale from 1 = against to 5 = in favor)

5) Alternative 1: Mandate vaccination of poultry against avian influenza
6) Alternative 2: Ban free-range systems
7) Alternative 3: Reduce poultry density in the region
8) Alternative 4: Culling (with compensation) in case of infection

Question 13: For which target group/region is this applicable?

(1) Alternative 1: Mandate vaccination of poultry against avian influenza and (2) Alternative 2: Ban free-range systems (scale from 1 = not applicable to 5 = applicable)

9) All poultry/kept birds
10) Only professional companies
11) Only private individuals/hobbyists
12) Only in (waterfowl-rich) risk zones

Question 14: For which target group/region is this applicable?

(3) Alternative 3: Reduce poultry density in the region and (4) Alternative 4: Culling (with compensation) in case of infection (scale from 1 = not applicable to 5 = applicable)

13) All poultry/kept birds
14) Only professional companies
15) Only private individuals/hobbyists
16) Only in (waterfowl-rich) risk zones

Question 15: (a) Please indicate which of the following methods you would apply to reduce the risk of infection from wild (water)birds in free-range systems. (b) Please indicate to what extent the following methods would reduce the risk of infection from wild (water)birds in free-range systems. (scale from 1 = reduction to 5 = no reduction)

4) Dense vegetation
5) Deterrents (e.g., lasers, aversive sounds, scarecrows, other animals, etc.)
6) Other

Survey professional laying hen farmers

Dear Egg-Laying Hen Farmer,

This survey is being sent to all egg-laying hen farmers in Flanders. On behalf of the Flemish Government, egg-laying hen farmers are surveyed about the usefulness of, and alternatives to, the confinement requirement for poultry to reduce the risk of infection and spread of avian flu in Flanders.

This survey is particularly useful for the Flemish egg-laying hen sector, allowing you to be heard by policymakers at both the regional and EU levels. It is also important for ILVO and other Flemish research institutions to conduct poultry research that aligns with the current concerns of the sector. To ensure the data is representative of the sector, all egg-laying hen farmers must participate in the survey. Only a large response will yield accurate and representative information for the sector. You would greatly help the sector and us by completing this survey.

In this survey, there are no right or wrong answers; what matters is your opinion. Naturally, we assure you that all information will be treated anonymously and confidentially; data from individual companies will not be disclosed, and the data will not be shared with third parties. All information is used solely for research purposes.

Question 1: What is your age?

- < 18 years
- 18 – 25 years
- 25 – 35 years
- 35 – 45 years
- 45 – 55 years
- 55 – 65 years
- > 65 years

Question 2: Please rate the following measures for the entire Belgian territory according to (scale from 1 = no compliance/not effective to 5 = full compliance/most effective)

(a) how meaningful/effective do you find them

(b) whether you have complied with them.

1) Cleaning (with approved biocide) or single-use of any vehicle and equipment used for transporting poultry, other captive birds, hatching eggs, or consumption eggs (= cleaning vehicle)

2) Investigation of any disease or abnormal mortality in poultry must be conducted immediately by the farm veterinarian or a certified veterinarian (= research disease).

3) Ban on access for any vehicle, person, and all materials to all places where poultry or other captive birds are kept if, in the past 4 days: they had contact with poultry or other captive birds in a risk area; they were in a risk area with poultry or other captive birds (= prohibition access from risk area)

4) Cleaning (with approved biocide) or single-use of any vehicle and equipment used for transporting poultry, other captive birds, hatching eggs, and consumption eggs in a third country or a risk area located outside Belgium (= cleaning vehicle abroad)

5) Ban on the trade of poultry and hobby poultry to and from hobbyists, including through markets (= prohibition trade of animals).

Question 3: Please rate the following measures for the entire Belgian territory according to (scale from 1 = no compliance to 5 = full compliance)

(c) whether you think fellow poultry farmers have complied with them,

(d) whether individuals/hobbyists have complied with them.

1) Cleaning (with approved biocide) or single-use of any vehicle and equipment used for transporting poultry, other captive birds, hatching eggs, or consumption eggs (= cleaning vehicle)

2) Investigation of any disease or abnormal mortality in poultry must be conducted immediately by the farm veterinarian or a certified veterinarian (= research disease).

3) Ban on access for any vehicle, person, and all materials to all places where poultry or other captive birds are kept if, in the past 4 days: they had contact with poultry or other captive birds in a risk area; they were in a risk area with poultry or other captive birds (= prohibition access from risk area)

4) Cleaning (with approved biocide) or single-use of any vehicle and equipment used for transporting poultry, other captive birds, hatching eggs, and consumption eggs in a third country or a risk area located outside Belgium (= cleaning vehicle abroad)

5) Ban on the trade of poultry and hobby poultry to and from hobbyists, including through markets (= prohibition trade of animals).

Question 4: Please rate the following measures at the level of individual poultry farms according to (scale from 1 = no compliance/not effective to 5 = full compliance/most effective)

(a) how meaningful/effective you find them,

(b) whether you have complied with them.

1) Placement of a disinfection footbath (with approved biocide) at the entrances and exits of each poultry shed (= disinfection bath).

2) Maintain a visit log with details (date, time of visit, name, address, license plate, reason, entry into sheds by the visitor) in chronological order. Each visit must be signed and dated by the farm veterinarian (= registration visitors).

3) Compulsory confinement of poultry or other captive birds to prevent contact with wild birds by keeping them indoors or by

4) Covering outdoor ranges with nets (mesh size of max. 10 cm)

5) Obligation to feed and water poultry and other captive birds indoors or by other means that prevent contact with wild birds

6) A ban on feeding or providing water to poultry and other captive birds from surface water supplies or rainwater to which wild birds have access unless the water has been treated to inactivate viruses

Question 5: Please rate the following measures at the level of individual poultry farms according to (scale from 1 = no compliance to 5 = full compliance)

(c) whether you think fellow poultry farmers have complied with them,

(d) whether individuals/hobbyists have complied with them.

1) Placement of a disinfection footbath (with approved biocide) at the entrances and exits of each poultry shed (= disinfection bath).

2) Maintain a visit log with details (date, time of visit, name, address, license plate, reason, entry into sheds by the visitor) in chronological order. Each visit must be signed and dated by the farm veterinarian (= registration visitors).

3) Compulsory confinement of poultry or other captive birds to prevent contact with wild birds by keeping them indoors or by

4) Covering outdoor ranges with nets (mesh size of max. 10 cm)

5) Obligation to feed and water poultry and other captive birds indoors or by other means that prevent contact with wild birds

6) A ban on feeding or providing water to poultry and other captive birds from surface water supplies or rainwater to which wild birds have access unless the water has been treated to inactivate viruses

Question 6: To what extent did the confinement had a negative or positive effect on… to (scale from 1 = negative to 5 = positive)

1) Workload
2) Productivity of hens
3) Income
4) Welfare of your poultry

Question 7: To what extent did the following animal welfare problems decrease or increase during the confinement obligation? (scale from 1 = decrease to 5 = increase)

1) Prevention of diseases
2) Feather pecking
3) Cannibalism
4) Stress and frustration in poultry
5) Red mite
6) Mortality in poultry
7) Workload
8) Foot dermatitis, hock dermatitis, and other foot injuries
9) Number of out-of-nest eggs
10) Percentage of first-quality eggs

11) Eggshell contamination

Question 8: To what extent did the following animal welfare problems increase or decrease during the confinement obligation? (scale from 1 = deterioration to 5 = improvement)

7) Bedding quality
8) Indoor climate
9) Condition of feathers, comb, wattles, and body

Question 9: Do you have any other examples of effects on the welfare of poultry you keep? If so, which ones?

Question 10: How did you implement the confinement obligation?

Please fill in 'Enter your comments here:' with your reasons.

- Covering the free-range with nets
- Indoor confinement

Question 11: Do you, consider it good or bad that placing nets over the range was also permitted instead of confining the birds indoors? (scale from 1 = bad to 5 = good)

Question 12: How feasible do you think it is to install nets over the range for your poultry? (scale from 1 = not feasible to 5 = feasible)

Question 13: Are you in favor of or against the following alternative measures against avian influenza? (scale from 1 = against to 5 = in favor)

9) Alternative 1: Mandate vaccination of poultry against avian influenza
10) Alternative 2: Ban free-range systems
11) Alternative 3: Reduce poultry density in the region
12) Alternative 4: Culling (with compensation) in case of infection

Question 14: For which target group/region is this applicable?

(1) Alternative 1: Mandate vaccination of poultry against avian influenza and (2) Alternative 2: Ban free-range systems (scale from 1 = not applicable to 5 = applicable)

17) All poultry/kept birds
18) Only professional companies
19) Only private individuals/hobbyists
20) Only in (waterfowl-rich) risk zones

Question 15: For which target group/region is this applicable?

(3) Alternative 3: Reduce poultry density in the region and (4) Alternative 4: Culling (with compensation) in case of infection (scale from 1 = not applicable to 5 = applicable)

21) All poultry/kept birds
22) Only professional companies
23) Only private individuals/hobbyists
24) Only in (waterfowl-rich) risk zones

Question 16: (a) Please indicate which of the following methods you would apply to reduce the risk of infection from wild (water)birds in free-range systems. (b) Please indicate to what extent the following methods would reduce the risk of infection from wild (water)birds in free-range systems. (scale from 1 = reduction to 5 = no reduction)

7) Dense vegetation
8) Deterrents (e.g., lasers, aversive sounds, scarecrows, other animals, etc.)
9) Other
